# Tetrodotoxin and Its Analogues (TTXs) in the Food-Capture and Defense Organs of the Palaeonemertean *Cephalothrix* cf. *simula*

**DOI:** 10.3390/toxins16010043

**Published:** 2024-01-12

**Authors:** Grigorii V. Malykin, Peter V. Velansky, Timur Yu. Magarlamov

**Affiliations:** A.V. Zhirmunsky National Scientific Center of Marine Biology, Far Eastern Branch, Russian Academy of Sciences, 690041 Vladivostok, Russia

**Keywords:** tetrodotoxin, tetrodotoxin analogues, TTX, HPLC–MS/MS, nemertea, buccal cavity, cephalic gland, proboscis

## Abstract

Tetrodotoxin (TTX), an extremely potent low-molecular-weight neurotoxin, is widespread among marine animals including ribbon worms (Nemertea). Previously, studies on the highly toxic palaeonemertean *Cephalothrix* cf. *simula* showed that toxin-positive structures are present all over its body and are mainly associated with glandular cells and epithelial tissues. The highest TTXs concentrations were detected in a total extract from the intestine of the anterior part of the body and also in a total extract from the proboscis. However, many questions as to the TTXs distribution in the organs of the anterior part of the worm’s body and the functions of the toxins in these organs are still unanswered. In the present report, we provide additional results of a detailed and comprehensive analysis of TTXs distribution in the nemertean’s proboscis, buccal cavity, and cephalic gland using an integrated approach including high-performance liquid chromatography–tandem mass spectrometry (HPLC–MS/MS), confocal laser scanning microscopy with anti-TTX antibodies, light and electron microscopies, and observations of feeding behavior. For the proboscis, we have found a TTXs profile different from that characteristic of other organs and tissues. We have also shown for the first time that the major amount of TTXs is localized in the anterior part of the proboscis that is mainly involved in hunting. TTX-containing glandular cells, which can be involved in the prey immobilization, have been found in the buccal cavities of the nemerteans. A significant contribution of the cephalic gland to the toxicity of this animal has been shown for the first time, and the role of the gland is hypothesized to be involved not only in protection against potential enemies but also in immobilizing prey. The data obtained have made it possible to extend the understanding of the role and features of the use of TTXs in the organs of the anterior part of nemertean’s body.

## 1. Introduction

The marine ribbon worm *Cephalothrix simula* is one of the most common representatives of the fauna from Asian coastal waters of the Northeast Pacific [1] and, since recently, from the Atlantic and Mediterranean coasts of Europe and the Pacific coast of North America [1,2,3]. A team of Japanese researchers led by Noguchi [4] obtained the first data on the toxicity of this nemertean species. In their analysis of approximately 200 specimens of *C. simula* (in the cited study referred to as *C. linearis* [1]), all of the specimens had strong paralytic toxicity. After it was proven in 2000 and 2003 that TTXs explain the major part of paralytic toxicity in representatives of *C. simula*, researchers in different fields showed increasing interest in this species [5,6]. In the subsequent years, studies were carried out not only on the taxonomy, geographical distribution, and general biology but also on various aspects of its toxicity. A genetic research has revealed three independent cryptic species hidden under the name *C. simula* [1,7]. One of these cryptic species, known as *Cephalothrix* cf. *simula*, is considered in the present study.

The function of this toxin in the phylum Nemertea was hypothesized as early as in the pioneer study based on detection of TTX in total extracts from the palaeonemertean *Tubulanus punctatus* and the heteronemertean *Lineus fuscoviridis* [8]. It was assumed to be a substance for (1) defense and (2) facilitating prey capture. However, data for *C. simula* (in the cited study referred to as *C. linearis* [1]) that confirmed the hypotheses about the biological role of TTX for nemerteans was obtained only two years later [4]. The authors showed that the mucus enveloping the nemertean and its food-capture tool (proboscis) exerts a high neuroparalytic effect. They also showed that the whole body (without proboscis) has a much greater neurotoxic effect compared to that of proboscis and mucus. However, it still remained unclear as to what role the toxins play in the nemertean’s body. According to the study of the intra-tissue distribution of the toxin, TTX is associated with gland cells of the epidermis and glandular epithelium of the proboscis, ciliated cells of the epidermis, enterocytes, and terminal cells of protonephridia [9,10]. It turned out that the amount of TTX in the nemertean’s body is distributed extremely unevenly: the major part of the toxin was detected in the total extract of the intestine from the anterior part of the body and the proboscis and then sharply decreased towards the tail. However, it is worth noting that the anterior part of the nemertean’s body contains various organs (cephalic gland, buccal cavity, and foregut) [11,12,13,14,15,16], and the proboscis is a morphologically heterogeneous structure consisting of several segments with different functions [13,16,17,18]. Therefore, despite the above-mentioned studies, such issues as the contribution of each organ in the anterior part of the worm’s body to the toxicity and details of TTXs distribution in the proboscis have been poorly understood to date.

In the present report, we provide results of a detailed and comprehensive analysis of TTXs distribution in the nemertean’s proboscis, buccal cavity, and cephalic gland using physicochemical, immunohistochemical, and morphological methods. The data obtained have made it possible to clarify the functional role of TTXs in these organs.

## 2. Results

### 2.1. General Results of HPLC–MS/MS

Concentrations of TTX and its analogues in a total extract of the proboscis, three regions of internal organs (hereinafter referred to as “intestine”), and the body wall were described in a previous study [19]. Here, we divided the proboscis of each of the examined nemerteans into 10 equivalent parts, and the internal organs (viscera) and the body wall into 11 to 15 parts. A total of 142 samples of tissues were analyzed (from 31 to 40 samples per individual) for the presence of TTX and its analogues (Supplementary Table S1). 

**Figure 1:**
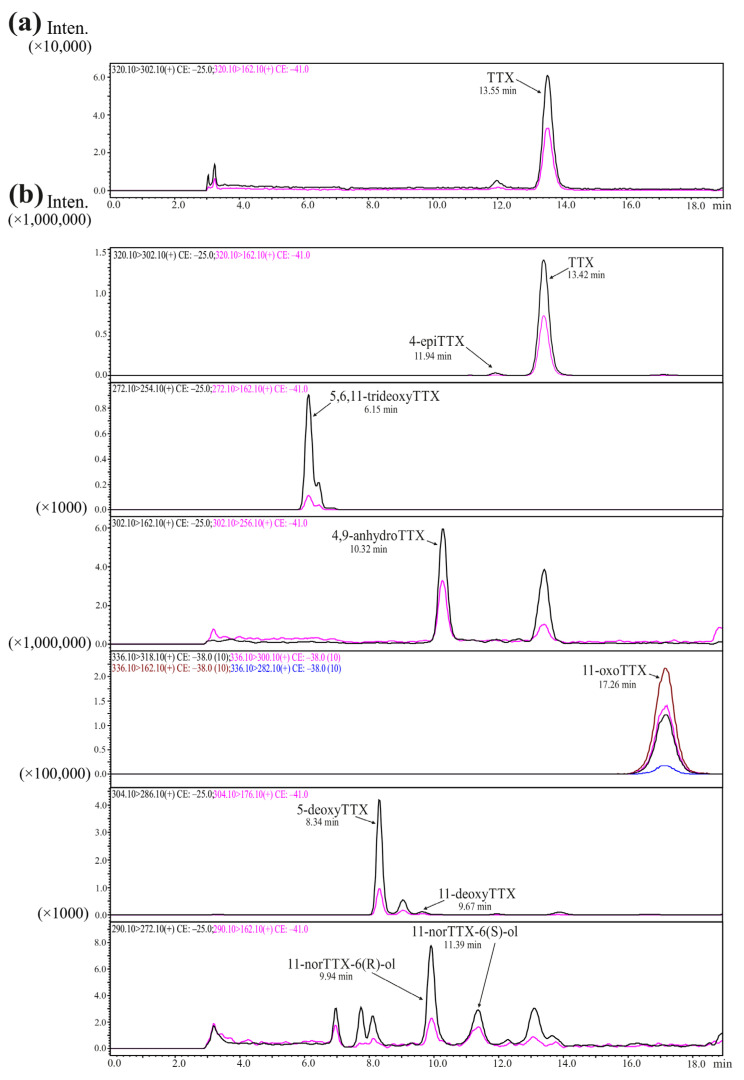
Chromatograms of tetrodotoxin and its analogues (TTXs) obtained by high-performance liquid chromatography–tandem mass spectrometry (HPLC–MS/MS). (**a**) TTX standard; (**b**) TTX and its analogues from extracts of *Cephalothrix* cf. *simula*. The curves of different colors represent different mass transitions (described in each chromatogram).

We identified from six to nine toxins in each of the samples analyzed: TTX, 4-epiTTX, 11-norTTX-6(s)-ol, 11-norTTX-6(r)-ol 4,9-anhydroTTX, 11-deoxyTTX, 5-deoxyTTX, 5,6,11-trideoxyTTX, and 11-oxoTTX (Table 1; Figure 1).

The TTXs in the individuals of *C*. cf. *simula* varied from the lowest amount of 2030.7 ng TTXs (individual 3) to a maximum of 172,836.7 ng (individual 1) (Table 1).

### 2.2. Body (Without Proboscis)

In the bodies of individuals 1 and 2, TTX and 5,6,11-trideoxyTTX proved to be the major toxins which made up, respectively, 96.5 and 89.2% of total toxins; the remaining seven analogues, that constituted 3.5 and 10.8%, respectively, were minor (Table 1). In the bodies of individuals 3 and 4, the major toxins were TTX, 5-deoxyTTX, and 5,6,11-trideoxyTTX, which, in total, made up 87.3 and 90.4%, respectively.

We did not find significant differences in the TTXs profile between the body fragments within each individual. There were also no significant differences in the profile of toxins between the body wall and the internal organs.

### 2.3. Proboscis

**Figure 2:**
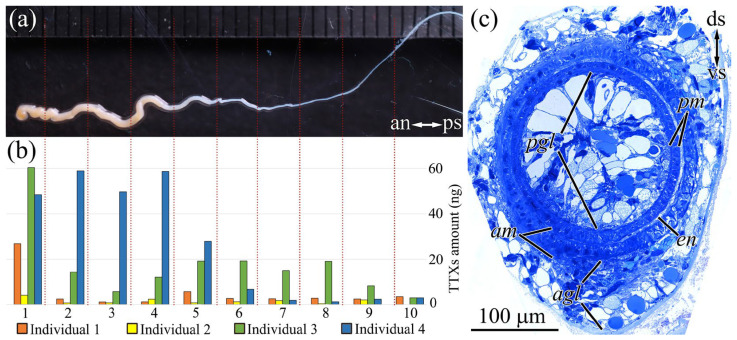
Schematic illustration of distribution of tetrodotoxin and its analogues (TTXs) in the proboscis of *C*. cf. *simula*. (**a**) Everted proboscis. Red lines indicate regions of cutting. (**b**) Amount of TTXs in 10 different sections of *Cephalothrix* cf. *simula* proboscis. (**c**) Light micrograph of a transverse section through the partially everted proboscis shows thick epithelium on the ventral side and thin epithelium on the dorsal side. *agl*, glandular epithelium of anterior proboscis segment; *am*, musculature of anterior proboscis segment; an, anterior side; ds, dorsal side; *en*, endothelium; *pgl*, glandular epithelium of posterior proboscis segment; *pm*, musculature of posterior proboscis segment; ps, posterior side; vs, ventral side.

The fully everted proboscis of a nemertean was visually divided into two parts: a thicker (with a diameter of up to 1.2 mm) anterior segment that constituted from 1/3 to 1/2 of the total proboscis length and a thin (of about 0.2 mm in diameter) posterior segment (Figure 2a). The proboscis length was 10–15 cm, which constituted approximately 2/3 of the worm’s total body length. In transverse histological sections, the everted proboscis appeared as a hollow muscular tube lined by flat endothelium inside and glandular epithelium outside. The glandular epithelium of the anterior proboscis had a height of up to 20 µm on the dorsal side and up to 100 µm on the ventral side; it consisted mainly of glandular cells with supportive cells scattered between them (Figure 2c) (details of cell composition of the anterior proboscis were considered by Malykin et al. [10]). The posterior part of the proboscis is not everted during hunting but remains inside the cavity of the anterior part of the everted proboscis. The thickness of the glandular epithelium on the dorsal and ventral sides of the posterior proboscis was approximately the same, i.e., no greater than 10–15 µm (Figure 2c). The cell composition of the glandular epithelium in the posterior part of the proboscis, as in the anterior part, was represented mainly by glandular cells with supportive cells evenly scattered among them. The thickness of the muscle layer and endothelium gradually decreased from 10–15 to 5–10 µm from the anterior to the posterior segments (Figure 2c).

The TTXs amount in the proboscises of the *C*. cf. *simula* individuals varied; the minimum amount was detected in individual 2 (19.5 ng), and the maximum amount was detected in individual 4 (391.3 ng). We identified from eight to nine TTXs in the extracts of proboscises (Table 1).

In all the analyzed individuals, the TTXs amount decreased from the anterior to posterior proboscis. In individuals 1, 2, and 3, the tip of the proboscis contained the greatest level of toxins (32.3–42.9%); in individual 4, about 80% was found in the first four fragments (18–22% per fragment) (Figure 2b).

TTX and 5,6,11-trideoxyTTX were present in the proboscises of all the analyzed individuals, where these toxins ranged within 22.9–55.6% and 11.1–28.6%, respectively. In individuals 1, 3, and 4, 5-deoxyTTX constituted a substantial proportion (16.9–19.1%). 11-oxoTTX, with ranges from 8.3 to 42.6%, was detected in individuals 1, 2, and 3. The total content of minor analogues was 3.2–14.5%. There were no significant differences in the TTXs profile between different proboscis fragments.

### 2.4. Buccal Cavity

**Figure 3:**
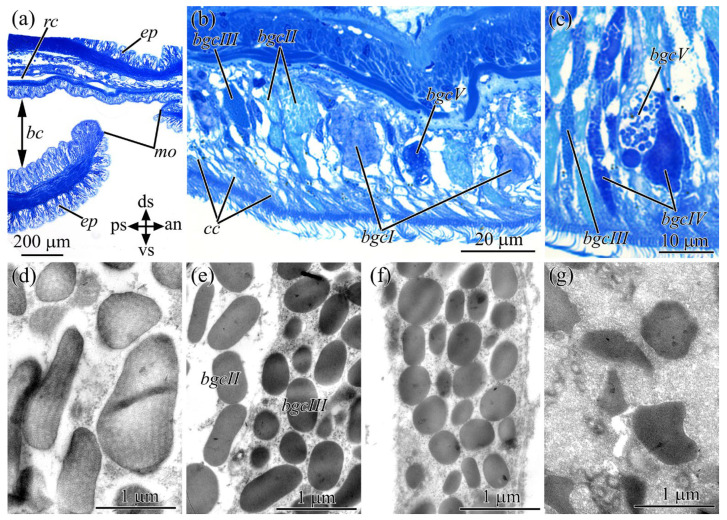
Light (**a**–**c**) and transmission electron (**d**–**g**) micrographs of sample sections, highlighting cell types of buccal cavity in *Cephalothrix* cf. *simula.* (**a**) General view of buccal cavity. (**b**,**c**) Buccal cavity epithelium with different cell types. (**d**) Secretory granules of type I glandular cells. (**e**) Secretory granules of types II and III glandular cells. (**f**) Secretory granules of type IV glandular cells. (**g**) Secretory granules of type V glandular cells. an, anterior side; *ep*, epidermis; *bc*, buccal cavity; *bgc I*, type I glandular cells; *bgc II*, type II glandular cells; *bgc III*, type III glandular cells; *bgc IV*, type IV glandular cells; *bgc V*, type V glandular cells; *cc*, ciliated cell; ds, dorsal side; *mo*, mouth; ps, posterior side; *rc*, rhynchocoel; vs, ventral side.

**Figure 4:**
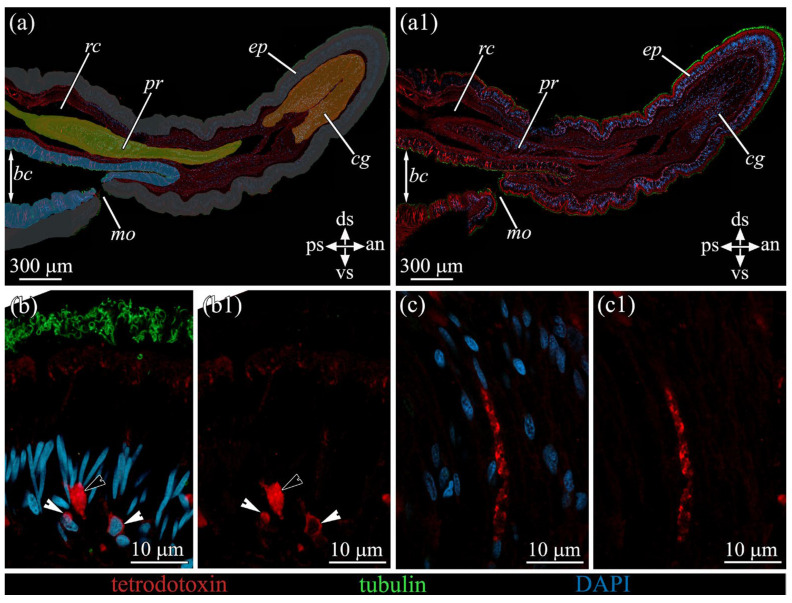
Tetrodotoxin-like immunoreactivity in the anterior part of *Cephalothrix* cf. *simula*. The CLSM micrographs show substacks of longitudinal sections. Red color indicates TTX-like immunoreactivity; green, α-acetylated tubulin immunoreactivity; blue, nuclei (DAPI). (**a**,**a1**) Panoramic view showing different organs: buccal cavity (blue color), proboscis (yellow), cephalic gland (orange), and epidermis (grey). (**b**,**b1**) TTX-positive granules of type I glandular cell (black arrowhead) and basal cells with TTX-positive perinuclear region (white arrowheads). (**c**,**c1**) TTX-positive granules of type V glandular cells. an, anterior side; *bc*, buccal cavity; *cg*, cephalic gland; ds, dorsal side; *ep*, epidermis; *mo*, mouth; *pr*, proboscis; ps, posterior side; *rc*, rhynchocoel; vs, ventral side.

In *C*. sf. *simula*, the mouth was located on the ventral side immediately behind the brain and opened into the buccal cavity (Figure 3a). The region including the buccal cavity occupied approximately 1/11 to 1/15 of the worm’s body length. The buccal cavity was lined with pseudostratified ciliary epithelium (Figure 3a–c and Figure 4a,b). Ciliated cells and five types of glandular cells were identified in the epithelium. In the basal part of the epithelium, basal (presumably low-differentiated) cells were distinguished that contained a large nucleus and a thin rim of perinuclear cytoplasm (Figure 4b). The ciliated cells were the most represented cell type. Large numbers of microvilli and cilia were evenly distributed over the apical surface of the cells (Figure 3b). Glandular cells differed in the shape and size of secretory granules and also in their contents. They were distributed evenly over the epithelium and occurred between ciliated cells (Figure 3b,c and Figure 4b,c). Type I glandular cells had oval-shaped secretory granules with angular edges (about 0.84 µm long and 0.48 µm wide) (Figure 3b,d). Contents of granules were a paracrystalline material consisting of fibrils of high or medium electron density embedded in a homogeneous material of low electron density. Type II glandular cells contained granules that varied from oval-mto rod-shaped, 0.8–1.3 µm in length and 0.22–0.6 µm in width (Figure 3b,e). The inner content of secretory granules was represented by a homogeneous material with medium electron density. Type III glandular cells contained rounded or oval granules of about 0.35 µm in length and 0.17 µm in width, with a homogeneous material of high electron density (Figure 3b,c,e). Type IV glandular cells contained rounded or oval granules of medium electron density, reaching 0.49 µm in length and 0.3 µm in width (Figure 3c,f). Type V glandular cells contained large, globe-shaped secretory granules (up to 1.6 µm in diameter) (Figure 3b,c,g). These granules had a heterogeneous material with a darkly stained and centrally located core embedded in densely packed fibrous matrix.

By way of the CLSM method using anti-TTX antibodies, an intensive TTX-positive labeling was found in basal low-differentiated cells and in types I and V glandular cells. In basal cells, a TTX-positive reaction was observed in thin semicircle-shaped or ring-shaped structures localized in the perinuclear area (Figure 4b). In type I glandular cells, a TTX-positive reaction was detected in secretory granules (Figure 4b); in type V glandular cells, on the periphery of secretory granules, with the central parts of the granules remaining non-stained (Figure 4c).

The profile of toxins in the buccal cavity epithelium was identical to that in the body. The proportion of toxins in the buccal cavity relative to that in all viscera amounted to 4.2, 5.18, and 7.4% in individuals 2, 3, and 4, respectively. 

In individual 1, the total proportion of toxins in the fragment including the entire precerebral region (“head”) and the buccal cavity relative to that in all viscera was 3.1%. 

### 2.5. Cephalic Gland

The cell types and morphology of the cephalic gland were previously described in the study by Malykin et al. [10], which showed that only one (mucoid cells) out of the three types of glandular cells contained TTX. The precerebral region analyzed in individuals 2 and 4 occupied, respectively, 1/11 and 1/15 of the total body length. The profile of toxins in the nemertean’s cephalic gland was identical to that in the body. The total amount of all TTXs in the cephalic gland relative to that in all intestines of the body was 3.0 and 17.9% in individuals 2 and 4, respectively. 

### 2.6. In Vivo Feeding Behavior

In all 10 cases of observation, the nemerteans, when hunting polychaetes, used their proboscises to immobilize prey. As time-lapse photography showed, the everted proboscis first moved straight along the prey’s body and then twisted around it, forming one to five coils. The neuroparalytic effect on the prey was manifested within 3–5 s. After 15–45 s, the prey with such a “garrote” around its body stopped moving (Supplementary Video S1). After a while, the proboscis holding the immobilized prey was retracted into the rhynchodaeum, and, simultaneously, the nemertean pulled itself up to the prey’s body. The nemertean crawled up onto the immobilized prey and began moving along its body continuously palpating the prey with its mouth. After finding a V-shaped bend or reaching one of the ends of the prey’s body, it started the ingestion process (Supplementary Video S2). Although the parapodia of the polychaete being ingested by the nemertean might partially retain their movements, when the prey’s body entered the digestive system, it was completely immobilized (Supplementary Video S3).

Three nemerteans that had lost their proboscis also showed the hunting behavior. In those cases, their prey items were either weakly mobile or resected polychaetes (Supplementary Video S4). As in the case of hunting with proboscis, polychaete’s parapodia completely stopped their movements after entering the nemertean’s digestive system.

## 3. Discussion

Studies of TTX-bearing marine organisms are largely fragmentary and, in most cases focus, on identification of new TTX-bearing species or analysis of commercial or conditionally commercial species for the presence of toxins [20,21,22,23,24,25,26]. Studies of the phenomenon of TTX accumulation by certain aquatic species are based only on pufferfish and consider seasonal variations of the toxin and its analogues in natural populations, identify mechanisms of directed transport of the toxin inside the body to the target organs, and search for TTX producers in the associated microflora [27,28,29,30,31,32,33,34]. However, the relatively complex anatomical and functional organization of fish makes it challenging to use them as objects for detailed studies of the patterns of entry of the toxin into the body and the features of its accumulation and utilization. An alternative model object for such studies may be the highly toxic nemertean *C. simula* that, on the one hand, has a simpler structure than vertebrates, and on the other, possesses all the main organ systems (nervous, circulatory, excretory, digestive, etc.) [13,15,16]. Furthermore, animals of this species can be kept in captivity for quite a long time (a year or more), which facilitates experiments of various types and is of interest, in particular, for investigations into their lifestyle and behavioral features (our observations). In the present study, we observed the behavior of *C*. Cf. *Simula* during hunting, which allowed us to extend the knowledge of the functional role of the TTX-bearing organs in the anterior part of the nemertean’s body and identify the features of their use.

The total TTXs content of the proboscis in the nemerteans that we examined varied greatly and differed between individuals by one or two orders of magnitude. The total level of toxins in the proboscis relative to that in the entire body of each individual also varied by two orders of magnitude and did not depend on the total amount of toxins in the worm (Table 1). The marked difference in the TTX content between proboscises of different individuals is likely explained by the use of the proboscis as a kind of “expendable” organ that can easily be damaged many times in the life and be partially or completely torn off during hunting, and then regenerated [17]. As our observations have shown, after being resected, it was capable of a quite rapid regeneration (within 5–7 days). The recovery of the formerly high TTX content in the organ that has lost the accumulated toxin is slow, as evidenced by a number of studies. Thus, in the Planocerid sp.1 flatworm, the level of TTXs in the pharynx is restored only within a few days after hunting [35]. As was reported for the heteronemertean *Kulikovia alborostrata*, the complete recovery of the TTXs level in the mucus secreted by the epidermis occurs within a month after the complete loss of toxins [36]. In newts, the regenerating skin at nine months post-injury usually contains markedly less TTX compared to the initial level [37]. It is worth noting here that the structures where the toxin depletion has been recorded may differ not only by a lower concentration of toxins relative to the initial one but also in the profile. Thus, in *K. alborostrata*, the accumulation of various analogues in mucus occurs at different rates after the complete depletion of TTXs in it [36]. However, not only the rate but also the pattern of accumulation of toxins may vary between different organs of animals. For instance, when several pufferfish species were fed toxin-containing food, different toxins accumulated in different organs such as liver, skin, intestines, and ovaries [28,32]. In our study, we did not find differences in the profile of toxins between the internal organs and the body wall of nemerteans. However, the unique toxin profile of the proboscis suggests a mechanism of TTXs accumulation here different from that in other organs and tissues. The different patterns of toxin accumulation may also be associated with different absorption rates of the analogues since they differ in physicochemical properties and/or there are various mechanisms of TTX transport specific to certain analogues. For example, the existence of a specific mechanism of TTX transport was reported for pufferfish in an experiment involving the incubation of sections of fish liver tissues in a toxin solution [38]. However, the most likely explanation for the accumulation of various analogues in the proboscis is the anatomical features of nemerteans; while the route of TTXs migration from the internal organs, including the intestinal epithelium, into the body wall does not encounter barriers, TTXs migrate into the proboscis through the rhynchodaeum system [9]. Such a system in *C*. cf. *simula*, as well as in all nemerteans, consists of the epithelium lining the rhynchodaeum, the cavity proper, and the endothelium surrounding the proboscis [15]. Such a multi-stage filtration of the toxin through the epithelial structures probably leads to a different accumulation pattern of different analogues in the nemertean proboscis. 

Previous studies of TTX localization in *C*. cf. *simula* using immunocytochemistry methods detected a weak fluorescent label in the proboscis muscle wall and an intense label in type II glandular cells of glandular epithelium [10]. In the present study, different individuals of *C*. cf. *simula* showed a clear relationship between the thickness of the glandular epithelium in the anterior segment of the proboscis and the distribution of the toxin over the organ: in all the animals analyzed, the major portion of the toxin was localized in the anterior segment of the proboscis. All of the above facts indicate the glandular epithelium as a structure where TTX is mainly accumulated in the proboscis. It should be noted that the thickest and, consequently, the most toxic ventral side of the anterior segment of the proboscis is mainly involved in hunting. As our experiments have shown, only the ventral side of the proboscis anterior segment comes in close contact with prey, while the entire anterior segment twists around the prey forming a loop. The neuroparalytic effect on the prey is manifested within 3–5 s, and complete paralysis occurs within 15–45 s after the attack. Thus, the success of hunting for *C*. cf. *simula* is associated not only with the presence of TTXs in the proboscis but also with the animal’s ability to efficiently use those parts of the prey-capture organ where the largest amounts of the toxin are localized. 

TTXs were detected in the salivary glands of TTX-bearing blue-ringed octopuses and in the pharynx of flatworms, which, along with the features of hunting behavior, has led to a hypothesis concerning the role of the toxin from the salivary and digestive glands as an agent for immobilizing prey [35,39,40]. In a recent study of TTX distribution, *C*. cf. *simula* larvae were found to have TTX-positive glandular cells in the mouth gland (an anlage of the future adult’s buccal gland) which larva can use for hunting [41]. This suggested the presence of similar toxin-containing glandular cells in the buccal cavity epithelium also in adult individuals. However, the buccal cavity was not mentioned in the studies on the localization of TTX in the digestive system of adult nemerteans [9,10], and no TTX-positive glandular cells were found in the examined regions (foregut and intestine) as well. In the present study, we have identified two types of TTX-positive glandular cells (types I and V buccal glandular cells) in the mouth and buccal cavity area of adult nemerteans *C.* cf. *simula* by immunohistochemical methods. Although the size of the buccal region relative to the total length of the digestive tract is not as significant (approximately 1/20 of the total digestive tract length), it accounts for up to 10% of TTXs in all internal organs. The involvement of these glandular cells in hunting is confirmed by our observations of this process in nemerteans: the prey becomes completely immobilized immediately after entering the nemertean’s buccal cavity, and then this immobilized prey is conveyed without delays into the foregut. Thus, the data we have obtained indicate that the toxin localized in the glandular cells of the buccal cavity can be used by nemerteans to immobilize prey. 

The cephalic gland of *C*. Cf. *simula* is an organ producing mucous secretion to the body surface in nemerteans. Such a secretion performs the function of mechanical protection against solid particles, facilitates movement over the bottom [11,14,42], and presumably serves to deter potential predators due to the presence of various toxins including TTX in it [4,19,43]. Studies of the TTX localization in the cephalic gland of *C*. cf. *simula* have identified two types of cells capable of accumulating TTX [10]. However, the contribution that the cephalic gland makes to the venomous characteristics of this animal has remained poorly understood to date. As our study shows, the cephalic gland can contain up to 41.4% of all toxins in the body. This organ occupies approximately 1/4 of the total region area, while the region proper constitutes approximately 1/20 of the total body length. The data obtained allows for an assumption that the cephalic gland is a very toxic organ, whose contribution to the toxicity of the mucus covering the nemertean’s body is quite substantial. The mucosal toxin-containing secretion is released through cephalic gland ducts that open on the surface of epidermis in the anterior part of the body [12]. It is worth noting that this part of the nemertean’s body comes in quite close contact with prey during hunting [44]. This suggests the involvement of the toxic secretion from the cephalic gland in prey immobilization [19]. Thus, the cephalic gland can perform both a defensive function, by saturating the mucus that coats the nemertean’s body with the toxin, and a hunting one.

To conclude, the present study is the first detailed assessment of TTXs distribution in the food-capture and defense organs of *C*. cf. *simula*. This study shows that nemertean, taking into account the physiological characteristics and distribution of TTXs in its proboscis, buccal cavity, and cephalic gland, is capable of maximizing the use of the toxins contained in these organs to immobilize prey and/or protect itself against predators. Further investigations of the ecological role of TTXs in nemerteans should be aimed at collecting direct evidence of entry and the mechanisms of toxins’ effects on target animals. 

## 4. Materials and Methods

### 4.1. Nemertans Collection

**Figure 5:**
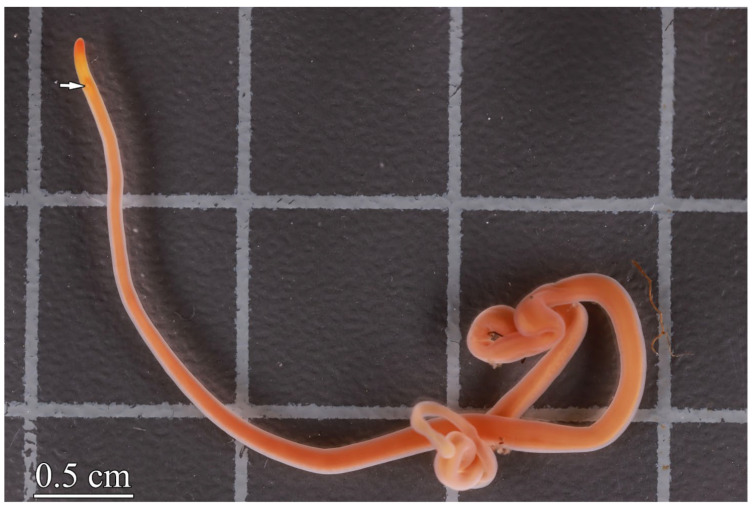
Live specimen of *Cephalothrix* cf. *simula*. Arrow indicates the mouth.

Taxonomical studies of *C. simula* have shown that this species comprises three networks [45] which are currently considered as independent cryptic species [1,7,46]. In the present study, the object was one of the cryptic species referred to as *Cephalothrix* cf. *simula* (Figure 5). All live ribbon worms were collected from Spokoynaya Bay (Sea of Japan) in July–August 2023 among brown algae (*Saccharina japonica*) rhizoids at a depth of 0.5 to 2 m. All individuals were identified to the species level by A.V. Chernyshev, an expert in nemertean systematics. The individuals were kept in separate tanks with seawater at 16–17 °C for 3 days before experiments. 

### 4.2. CLSM and Morphological Studies

For CLSM and morphological studies, two individuals each were used. Immunohistochemical procedures were carried out according to the standard protocol described by Malykin et al. [10]. In brief, live individuals of *C*. cf. *simula* were relaxed in a 7% solution of MgCl_2_, dissected into small pieces, and fixed with a paraformaldehyde solution (4.0%) in phosphate-buffered saline (PBS, pH 7.8). After being rinsed with PBS, the samples were embedded in 20% saccharose solution and cut into sections (approximately 10 μm thick) on a Thermo HM 560 cryotome (Thermo Fisher Scientific, Waltham, MA, USA). To visualize TTXs-positive structures, a solution of rabbit polyclonal anti-TTX antibodies (1:25, Genetex, Irvine, CA, USA) was used; to visualize tubulin-immunoreactive structures, mouth polyclonal anti-acetyl α-tubulin antibodies were used (1:1000, Sigma-Aldric, St. Louis, MO, USA). The sections were incubated in a solution of primary antibodies for 2 d at 4 °C, washed with PBS with Tween-20 (0.05%, Sigma-Aldric, St. Louis USA) (TBST). For immunoreactivity visualization, sections were incubated in solutions of Alexa Fluor 488 (1:500, Invitrogen, Waltham, MA, USA) and Alexa Fluor 647 (1:500 Invitrogen, Waltham, MA, USA) secondary antibodies for 1 d at 4 °C, and stained with 4′,6-diamidino-2-phenylindole (DAPI). The sections were embedded in Mowiol 4-88 (Sigma-Aldrich, St. Louis, MO, USA) and examined under LSM-780 microscope (Carl Zeiss, Jena, Germany). As a negative control, a non-immune rabbit serum and PBS with 10% bovine serum albumin were used. 

For morphological studies, the material was fixed in a 2.5% glutaraldehyde solution in PBS. Then, post-fixation was carried out in 1% OsO_4_. The material was gradually dehydrated in series of ethyl alcohol and acetone solutions. After that, the material was embedded in Epon-Araldite resin (EMS, Hatfield, PA, USA) and cut into thin (60 nm) and semithin sections (1 μm thick) on an Ultracut E ultramicrotome (Leica Biosystems, Wetzlar, Germany). The semithin sections were stained with methylene blue (Sigma, USA) and examined under a Zeiss Axio Imager Z2 microscope (Carl Zeiss, Jena, Germany). Thin sections were stained with 1% uranyl acetate and 0.35% lead citrate solutions and were examined under the Libra 120 transmission electron microscope (Carl Zeiss, Jena, Germany).

### 4.3. Extraction and Analysis of TTX and Its Analogues

For TTXs extraction and analysis, four individuals were used. The individuals were anesthetized in a 7% solution of magnesium chloride and fixed in 96% ethanol. After that, the proboscis was separated from the worm’s body and divided into 10 fragments equal in length. The body was divided into 11 to 15 equal-sized fragments depending on the size of the animal; in addition, each fragment was divided into the body wall and the internal organs. Then, the fragments were fixed in 0.5 mL 0.1% solution of acetic acid in 70% ethanol. The samples were homogenized in a 0.1% solution of acetic acid in 70% ethanol using a hand homogenizer. Then the samples were homogenized on an HD 2070 ultrasonic homogenizer (Bandelin Sonopuls, Berlin, Germany) for 10 min (frequency, 20 kHz; amplitude, 228 µm; working cycle, 0.8 s; and interval, 0.2 s). The samples were centrifuged (8000× *g*, 10 min, 4 °C). Supernatants were filtered with 3 kDa on a Vivaspin turbo centrifugal concentrators (Sartorius, Goettingen, Germany). The extracts were evaporated on a Labconco 7810030 vacuum concentrator (Labconco, Kansas City, MO, USA) at 60 °C. Then, the extracts were dissolved in 50 μL of a 0.1% aqueous solution of acetic acid.

For identification and quantification of TTXs, the high-performance liquid chromatography with tandem mass spectrometry (HPLC–MS/MS) was used according to Bane et al. [47] with modifications described by Vlasenko et al. [48] (Supplementary File S1). The limit of quantification (LoQ) was 0.6 ng/mL; the limit of detection (LoD), 0.2 ng/mL.

The concentration of TTXs was converted to their amount in tissues. The amount of toxins in each sample that corresponded to a value below LoD and LoQ was less than 0.02 and 0.06 ng, respectively.

### 4.4. Feeding Experiments

For feeding experiments, 10 individuals were used. To study the features of nemertean’s feeding behavior, they were placed, along with polychaetes *Dorvillea* sp. or their cut-off fragments, in Petri dishes containing seawater at 17 °C. To observe the feeding behavior of nemerteans that lack proboscis, in some of individuals, the proboscises were preliminarily cut off. Observations and video recordings were carried out using a Stemi 305 binocular stereomicroscope (Carl Zeiss, Jena, Germany) and a Canon EOS 6D Mark II camera (Canon Inc., Tokyo, Japan). Observations of each individual were carried out for 2–3 h.

## Figures and Tables

**Table 1:** Tetrodotoxin (TTX) and its analogues in different regions and organs of *Cephalothrix* cf. *simula*.

Individual No.	Weight, g	Sex	Region	Organ/Segment	Toxins														
					ng *												**%**		
					TTX	4-epiTTX	11-norTTX-6(r)-ol	11-norTTX-6(s)-ol	4,9-anhydroTTX	5-deoxyTTX	11-deoxyTTX	5,6,11-trideoxyTTX	11-oxoTTX	Total of TTXs	Total of TTXs in Regions	Total Amount of TTXs	**% of Amount in Region**	**% of TTXs in Organ/Section in Total Amount**	**% of TTXs in Region of Total Amount**
1	0.2	Female	Body	Internal organs	78,651.9	323.4	11.5	<0.02	203.9	1610.2	1160.4	30,903.6	10.2	112,875.2	167,216.9	172,836.7	67.5	65.3	96.7
				Body wall	38,724.7	270.2	3.2	<0.02	124.3	1077.8	639.5	13,501.4	0.6	54,341.7			32.5	31.4	
			Bc + Cg	Internal organs	2897.5	24.9	<0.02	1.4	9.8	148.5	27.1	515	8.9	3633	5533.1		65.7	2.1	3.2
				Body wall	1406.3	13.5	<0.02	<0.02	3.9	78.8	13.6	383.9	<0.02	1 900			34.3	1.1	
			Proboscis	Anterior segment	27.2	0.6	<0.02	0.7	0.1	6.5	0.3	13	6.4	54.8	86.8		63.1	0.0	0.1
				Posterior segment	10.4	0.4	<0.02	<0.06	<0.06	8.2	0.2	11.9	0.9	32			36.9	0.0	
2	0.07	Female	Body	Internal organs	1023.1	142.8	0.4	4.4	48.8	282.3	64.7	605.4	19.3	2191.1	16,230.8	19,162.0	13.5	11.4	84.7
				Body wall	7382.3	604.4	0.2	6.2	166.8	256.3	144.5	5478.9	<0.02	14,039.7			86.5	73.3	
			Bc	Internal organs	57.3	3.5	<0.02	0.1	1.1	4.5	1.2	30.6	1.7	100	2040.9		4.9	0.5	10.7
				Body wall	1123.2	55.2	<0.02	1.2	7.3	40.5	16.9	696.6	<0.02	1940.9			95.1	10.1	
			Cg	Cg	36.7	2	<0.02	0.2	0.6	4.5	0.8	25.5	<0.02	70.4	870.8		8.1	0.4	4.5
				Body wall	282.2	88.0	0.1	1.3	24.6	47.5	5.7	351.1	<0.02	800.4			91.9	4.2	
			Proboscis	Anterior segment	2.3	0.2	0.2	0.3	0.1	0.5	<0.06	4.5	5.2	13.3	19.5		66.3	0.1	0.1
				Posterior segment	1.8	0.1	<0.02	0.4	<0.02	0.3	<0.02	0.5	3.1	6.2			33.7	0.0	
3	0.09	Male	Body	Internal organs	393.4	20.2	0.2	2.6	4.4	195.5	31.9	85.1	11	744.4	1659	2030.7	44.9	36.7	81.7
				Body wall	238.6	43.2	0.4	3.3	11.1	293.2	79.8	245.1	<0.02	914.7			55.1	45.0	
			Bc	Internal organs	23.4	0.9	<0.02	0.1	0.2	8.9	1.6	4.7	0.8	40.6	130.2		31.2	2.0	6.4
				Body wall	21.5	4.1	<0.06	0.4	0.6	23	7.3	32.6	<0.02	89.6			68.8	4.4	
			Proboscis	Anterior segment	49.9	3.1	0.4	14	0.7	28.5	2.2	17.2	36.6	152.5	241.5		63.2	7.5	11.9
				Posterior segment	28.5	1.4	0.2	10.7	0.3	17.6	1.6	9.7	19	89			36.8	4.4	
4	0.11	Male	Body	Internal organs	715.3	37.8	1.3	0.8	6.6	617	24.3	240.7	1.4	1645	3314.1	4990.8	49.6	33.0	63.6
				Body wall	444.1	117.3	2.4	2	9	652.2	78.5	363.6	<0.02	1669.1			50.4	33.4	
			Bc	Internal organs	63.1	3.7	0.1	<0.06	0.6	48.7	2.4	44	<0.02	162.5	331.9		49	3.3	6.4
				Body wall	38	14.5	0.4	0.2	0.7	57.4	10.2	48	<0.02	169.3			51	3.4	
			Cg	Cg	199.3	22.4	0.4	0.7	8.7	91.1	3.7	68.2	<0.02	394.6	953.6		41.4	7.9	18.3
				Body wall	161.3	48.7	2.5	1.8	12.0	205.1	22.7	104.9	<0.02	559			58.6	11.2	
			Proboscis	Anterior segment	206.2	6.1	0.9	6.8	2.2	58.3	3.9	68.3	4.9	357.4	391.3		91.3	7.2	11.7
				Posterior segment	11.5	0.5	0.1	0.2	0.2	10.1	0.3	10.7	0.3	33.9			8.7	0.7	

* The amount of toxin reported is determined by summing up the individual amounts of TTX in a number of samples obtained from the corresponding individual. Bc: buccal cavity; Cg: cephalic gland.

## Data Availability

All data generated and analyzed in this study are available within the article and on the Figshare repository (https://figshare.com/, accessed on 4 December 2023): 10.6084/m9.figshare.24720039.

## Supplementary Materials

The following supporting information can be downloaded at https://www.mdpi.com/article/10.3390/toxins16010043/s1: Table S1: MRM transitions and collision energy for TTX and its analogues; Video S1; Video S2; Video S3; Video S4; File S1: Parameters of TTX and its analogues analysis. References [47,49,50,51,52,53,54] are cited in the supplementary materials.
