# Supplementary material for: Tetrodotoxin and Its Analogues (TTXs) in the Food-Capture and Defense Organs of the Palaeonemertean Cephalothrix cf. simula
Source: Toxins (Basel). 2024 Jan 12;16(1):43. doi: 10.3390/toxins16010043 (PMC10818845; doi:10.3390/toxins16010043)
Supplement: Supplementary file 1 [file toxins-16-00043-s001.zip › File S1.pdf]

Tetrodotoxin and Its Analogues (TTXs) in the Food-Capture and Defense Organs of the Palaeonemertean *Cephalothrix* cf. *simula*

Grigorii V. Malykin, Peter V. Velansky and Timur Yu. Magarlamov

Parameters of TTX and its analogues analysis

TTX and its analogues were identified using HPLC–MS/MS. The HPLC system included two pairs of LC-30 pumps, a SIL-30AC autosampler, a CTO-20A thermostat, an SCL-20A system controller, and a triple quadrupole mass spectrometer LCMS-8060 (Shimadzu Europa, Duisburg, Germany) with electrostatic spray ionization (ESI). Separation was carried out using a SeQuant ZIC HILIC column (150 × 2.1 mm, 5 μm) (Merck, Darmstadt, Germany) at 40 °C and a flow rate of 0.25 ml/min. A binary gradient was used: mobile phase A, ammonia (5 mM) and formic acid (8 mM) in 9:1 acetonitrile–water; mobile phase B, ammonia (10 mM) and formic acid (20 mM) in water. A gradient profile was used as follows: (a) 0–4.3 min, 15% B; (b) 4.4–17 min, 25% B; (c) 17–20 min, 50% B. The sample volume was 1 μL. A SeQuant ZIC-HILIC guard column (20 × 2.1 mm, 5 μm) (Merck, Darmstadt, Germany) was installed in line before the analytical column through a two-position 6-port valve. At 4.4 min, the valve was switched and guard columns backflashed with isopropanol (4.4–7 min) and water (7–14 min) at flow rate of 0.2 ml/min. At 17 min, the valve was switched back. The mass spectrometer was operated in scan (m/z 200–1000) and multiple reaction monitoring (MRM) modes. The ion source parameters were as follows: interface temperature—280°C, desolvation line temperature—250°C, nebulizing gas (N2) flow—3 L/min, drying gas (N2) flow—3 L/min, and heating gas (dry air) flow—17 L/min. Collision energy for the analyzed substances is indicated in Table 1. The TTX concentration was calculated using the calibration curve of a standard TTX solution series (Alomone Labs Ltd., Jerusalem, Israel). The toxin detection criteria included a precursor MRM transition peak S/N ratio > 3, relative intensity of the fragment ion peak > 4%, and the order of toxins elution corresponded to that described in Bane et al. [47]. MRM transitions were used to detect TTX analogues as described by Vale [49], Kudo et al. [50], Bane et al. [51], Puilingi et al. [52], and Turner et al. [53] and indicated in Table 1. The concentrations of TTX analogues were calculated following the procedure of Chen et al. [54]. The method was validated using standard TTX solutions in MRM mode. The linearity range was from 0.6 to 100 ng/mL. The recovery range from 1 to 100 ng/mL of TTX was 98.4%. The LoQ was 0.6 ng/mL. The limit of detection (LoD) was 0.2 ng/mL, and the relative SD was 4.5%–14.6%.

Table 1. MRM transitions and collision energy for TTX and its analogues.

№	Analogue	MRM transitions	Collision Energy (eV)
1	TTX	320.1>302.1	25, 38
		320.1>162.1	36.949–55.423
2	4-epiTTX	320.1>302.1<br>320.1>162.1	25, 38
3	4,9-anhydroTTX	302.1>256.1	30
		302.1>162.1	49
4	11-norTTX-6(R)-ol	290.1>272<br>290.1>162.1	30
5	11-norTTX-6(S)-ol	290/272	49
		290.1>162.1	30
6	11-deoxyTTX	304>286<br>304>176	49
7	5-deoxyTTX	304>286<br>304>176	49
8	5,6,11-trideoxyTTX	272.1>162.1<br>272.1>254.1<br>208.1064;<br>180.1137;<br>178.0954;<br>162.1020;<br>133.0724;	33.570–50.358
9	11-oxoTTX	336>318<br>336>300<br>336>282<br>336>178<br>336>162	38.1–57.1
